# Correction to: Tackling geographic barriers to primary dental care (dental deserts): a systematic review

**DOI:** 10.1038/s41415-025-9001-z

**Published:** 2025-07-25

**Authors:** James W. Edwards, Birke Bogale, Jennifer E. Gallagher

**Affiliations:** 176564349371022934750 DCT1 and Visiting Research Assistant, Dental Public Health, Faculty of Dentistry, Oral and Craniofacial Sciences, King´s College London, Bessemer Road, London, SE5 9RS, United Kingdom; 607761973503786621961 PhD Candidate in Dental Public Health, Faculty of Dentistry, Oral and Craniofacial Sciences, King´s College London, Bessemer Road, London, SE5 9RS, UK; Department of Dental and Maxillofacial Surgery, St Paul´s Hospital Millennium Medical College, Addis Ababa, Ethiopia; 141633907186324291937 Ambassador International, Engagement and Service, King´s College London, UK; Newland-Pedley Professor of Oral Health Strategy and Honorary Consultant in Dental Public Health, Faculty of Dentistry, Oral and Craniofacial Sciences, King´s College London, Bessemer Road, London, SE5 9RS, United Kingdom

Journal's correction note:

Research article *Br Dent J* 2025; DOI: 10.1038/s41415-025-8487-8

When initially published, Appendix 1 was missing from the online version of this article due to an XML error. This has since been corrected and Appendix 1 is present in the online version.

The journal apologises for any inconvenience caused.

